# The “Adipo-Muscular” association in heart failure with preserved ejection fraction: synergistic impact of epicardial adipose tissue and skeletal muscle endurance on functional capacity

**DOI:** 10.1186/s12872-026-05833-6

**Published:** 2026-04-22

**Authors:** Ertan Aydin, Habibe Durdu, Asli Vural, Ceren Yildirim, Devrim Kurt, Ulfat Bayramlı, Gökhan Gök

**Affiliations:** 1 Department of Cardiology, Faculty of Medicine, Giresun University, Giresun, Turkey; 2 Department of Therapy and Rehabilitation, Giresun University, Vocational School of HealthServices, Giresun, Turkey

**Keywords:** HFpEF, Epicardial Adipose Tissue, Myopenia, Muscle Endurance, EAT-Endurance Index, 6PBRT

## Abstract

**Background:**

Heart failure with preserved ejection fraction (HFpEF) is increasingly recognized as a systemic multiorgan syndrome driven by low-grade inflammation. While epicardial adipose tissue (EAT) and skeletal muscle dysfunction are known contributors to exercise intolerance, their synergistic association remains undefined.

**Aims:**

We aimed to investigate the “Adipo-Muscular” association—the synergistic interplay between local cardiac inflammatory potential and peripheral muscle fatigability—as a predictor of functional capacity in HFpEF.

**Methods:**

This cross-sectional study enrolled 192 stable HFpEF patients and 200 age- and sex-matched healthy controls. EAT thickness was quantified via echocardiography. Peripheral muscle function was assessed through maximal isometric strength and handgrip endurance (maintenance time at 50% maximal voluntary contraction), the latter serving as a surrogate for Type I fiber oxidative capacity. Functional capacity was validated using the 6-Minute Pegboard Ring Test (6PBRT), specifically chosen for its sensitivity to upper extremity “myopenia” (qualitative bioenergetic failure). A novel EAT-Endurance Index was calculated as: Index = ({EAT thickness [mm]} / {Handgrip endurance [sec]}) ×100.

**Results:**

Despite comparable BMI between groups, HFpEF patients displayed significantly greater EAT thickness (5.46 ± 1.65 vs. 3.20 ± 1.03 mm, *p* < 0.001). While maximal strength showed a moderate decline, handgrip endurance was profoundly compromised in HFpEF (48.48 ± 14.4 vs. 72.24 ± 12.1 s, *p* < 0.001), representing a massive effect size (Cohen’s d = -1.791). In multivariate analysis adjusting for age, sex, BMI, and major comorbidities (Hypertension, Diabetes, and Coronary Artery Disease), the EAT-Endurance Index emerged as the strongest independent predictor of impaired functional capacity (β = 0.385, *p* < 0.001). Sensitivity analysis confirmed this association was independent of glycemic status.

**Conclusions:**

We identified a distinct “Adipo-Muscular” phenotype in HFpEF, characterized by expanded epicardial fat and profound skeletal muscle fatigability. This association predicts functional decline independently of systemic obesity, maximal strength, and classic comorbidities. Targeting the interface between central adiposity and skeletal muscle mitochondrial function may offer novel therapeutic avenues for this challenging population.

## Introduction

The conceptual framework of Heart Failure with Preserved Ejection Fraction (HFpEF) has undergone a paradigm shift, evolving from a hemodynamic-centered view of diastolic dysfunction to the recognition of a systemic, multiorgan syndrome. The unifying theory proposed by Paulus and Tschöpe identifies systemic low-grade inflammation—driven by comorbidities—as the primary pathophysiological trigger [1, 2]. This inflammatory cascade leads to coronary microvascular endothelial dysfunction, cardiomyocyte stiffening, and interstitial fibrosis. However, while the downstream cardiac consequences are well-documented, the specific “generators” of this inflammation and their remote impact on peripheral exercise tolerance remain under active investigation.

In this complex landscape, Epicardial Adipose Tissue (EAT) has emerged not merely as a visceral fat depot, but as a biologically active endocrine organ [3]. Uniquely situated without a fascial barrier between EAT and the underlying myocardium. EAT shares the same microcirculation. In HFpEF, EAT undergoes a transformation into a pro-inflammatory phenotype, secreting cytokines (e.g., IL-1β, TNF-α) and adipokines via paracrine (“vasocrine”) signaling [3, 4]. This local inflammatory burden is strongly associated with adverse cardiac remodeling, independent of systemic obesity (BMI) [5].

Concurrently, exercise intolerance—the hallmark symptom of HFpEF—is often disproportionate to central hemodynamic aberrations, pointing towards peripheral limitations [6]. Skeletal muscle abnormalities in HFpEF extend beyond simple atrophy (sarcopenia). Recent evidence highlights qualitative defects (“myopenia”), including capillary rarefaction, fiber-type switching, and impaired mitochondrial oxidative phosphorylation [7]. Unlike sarcopenia, which focuses on muscle mass, myopenia refers to qualitative defects in muscle tissue, including mitochondrial dysfunction and impaired microvascular perfusion, which are hallmark features of the HFpEF peripheral phenotype. Critically, while maximal muscle strength largely reflects contractile protein mass, muscle endurance serves as a superior surrogate for mitochondrial bioenergetics and microvascular integrity. The inability to sustain submaximal effort (fatigability) may be more clinically relevant to the daily life limitations of HFpEF patients than peak strength alone [8]. 

Despite the established roles of these two compartments, the interplay between the “central” inflammatory potential source of local pro-inflammatory mediators (EAT) and the “peripheral” metabolic failure (muscle endurance) has not been fully elucidated. We hypothesize the existence of a deleterious adipo-muscular association, wherein inflammatory mediators postulated to originate from visceral cardiac adiposity may contribute to a systemic environment that exacerbates peripheral muscle fatigability possibly via the dysregulation of the adipomyokine network [9]. Therefore, this study aims to characterize this novel phenotype, postulating that the combination of high EAT burden and compromised muscle endurance represents a synergistic predictor of impaired functional capacity (measured by the 6-Minute Pegboard Ring Test (6PBRT) in patients with HFpEF.

## Materials and methods

### Study design and population

This observational, cross-sectional study was conducted at (Giresun Training and Research Hospital) between [06/02/2026] and [16 /03/2026]. The study protocol complied with the Declaration of Helsinki and was approved by the Institutional Review Board (protocol code 2026/21). All participants provided written informed consent.

We enrolled 192 consecutive patients with stable HFpEF and 200 age- and sex-matched healthy controls. HFpEF diagnosis adhered strictly to the 2021 ESC Guidelines on Acute and Chronic Heart Failure and the HFA-PEFF diagnostic algorithm [Pieske et al., 2019; DOI: 10.1093/eurheartj/ehz641] [8]: (i) presence of symptoms and/or signs of HF; (ii) preserved left ventricular ejection fraction (LVEF > 50%); and (iii) objective evidence of cardiac structural and/or functional abnormalities consistent with LV diastolic dysfunction/raised LV filling pressures, including Left Atrial Volume Index (LAVI) > 34 mL/m², Left Ventricular Mass Index (LVMI) > 115 g/m² (males) or > 95 g/m² (females), or an E/e’ ratio > 9.

Exclusion criteria were: acute coronary syndromes or revascularization within the last 3 months, significant valvular heart disease (moderate-to-severe stenosis or regurgitation), primary cardiomyopathies (hypertrophic, infiltrative), active malignancy, and primary musculoskeletal or neuromuscular disorders preventing handgrip testing. Healthy controls were recruited from subjects referred for cardiac screening who showed no evidence of cardiovascular disease.

### Echocardiographic & Epicardial Adipose Tissue (EAT) Quantification

Standard transthoracic echocardiography was performed using a high-end ultrasound system (Philips Affiniti 70, Philips Healthcare, Andover, MA, USA) equipped with a 2.5–3.5 MHz phased-array transducer, following current ASE/EACVI recommendations.

**Fig. 1:**
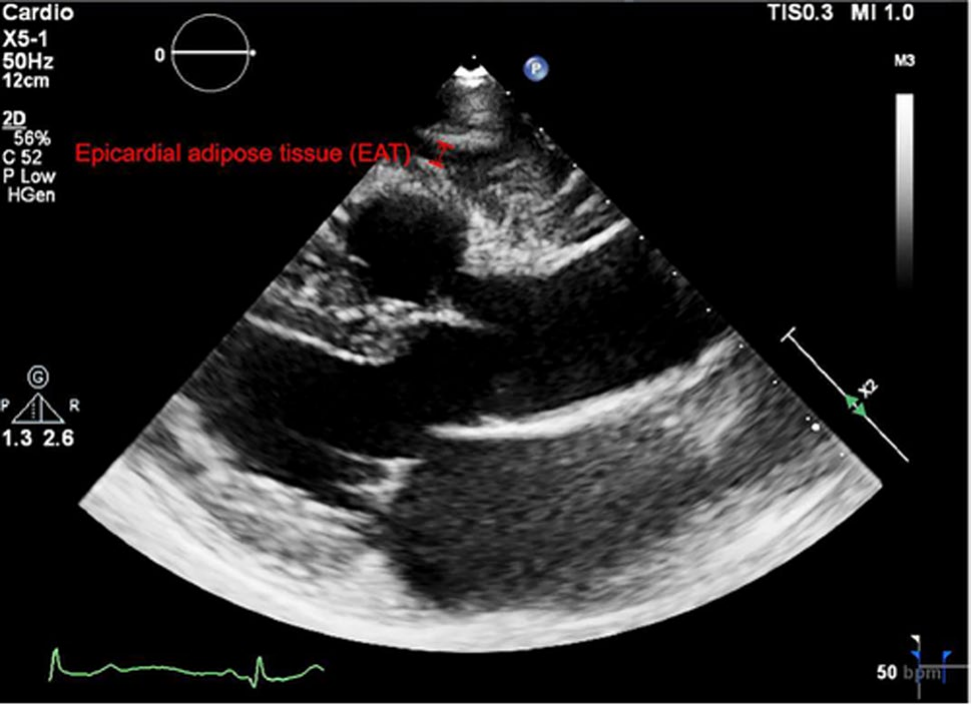
Standardized Assessment of Epicardial Adipose Tissue (EAT) Thickness using Transthoracic Echocardiography

EAT thickness was quantified in the parasternal long-axis view at end-systole (Fig. 1). The measurement was taken strictly perpendicular to the right ventricular free wall, identifying the maximal echo-free space between the myocardium and the visceral pericardium [originally described by Iacobellis et al., 2003; DOI: 10.1038/oby.2003.45] [10]. To ensure reliability, the average of three consecutive cardiac cycles was recorded. To assess reproducibility, EAT thickness was re-measured in a random subset of 20 patients by a second blinded observer, yielding an inter-observer correlation coefficient of 0.92. Additionally, the primary observer re-measured the same subset two weeks later, showing excellent intra-observer reproducibility with a correlation coefficient of 0.94.

Representative transthoracic echocardiographic image in the parasternal long-axis view demonstrating the standardized assessment of EAT thickness. The EAT is identified as the echo-lucent (hypoechoic) space situated between the visceral layer of the pericardium and the epicardium of the right ventricular (RV) free wall. The red arrow and label indicate the precise measurement point, which is taken perpendicularly to the RV free wall at the level of the aortic annulus during end-systole (defined by the end of the T-wave on the electrocardiogram). This standardized approach, relying on clear anatomical landmarks and cardiac cycle synchronization (EKG visible in the lower left), ensures maximal reproducibility and comparability across different cohorts.

### Assessment of peripheral muscle function

Muscle function tests were performed in a quiet room with the participant seated comfortably, shoulder adducted, and elbow flexed at 90° without arm support.

- Maximal Isometric Handgrip Strength: Assessed using a calibrated hydraulic hand dynamometer (Jamar, Bolingbrook, IL, USA). Participants performed three maximal voluntary contractions (MVC) with the dominant hand, separated by 60-second rest intervals. The highest value (kg) was recorded for analysis.
- Handgrip Endurance (skeletal muscle fatigue resistance test- a surrogate marker for Type I fiber oxidative capacity): This metric was utilized to assess local muscle fatigue resistance. After determining the MVC, participants were instructed to maintain a submaximal contraction at 50% of their specific MVC for as long as possible. Visual feedback was provided to help the participant maintain the target force. The test was terminated when the force dropped below the 50% threshold for > 3 s. The time to failure (seconds) was recorded as an index of Type I fiber oxidative capacity.

### Functional capacity assessment (6PBRT)

To provide a functional validation specifically aligned with upper extremity muscle performance, the 6-Minute Pegboard Ring Test (6PBRT) was utilized instead of traditional walking tests.Functional capacity regarding upper extremity activities of daily living was objectively assessed using the 6-Minute Pegboard Ring Test (6PBRT). Patients were instructed to move as many rings as possible from a container to pegs on a vertical board within 6 min. The total number of rings moved (score) was recorded.

### Statistical analysis

Statistical analyses were performed using SPSS Statistics for Windows, Version [e.g., 26.0] (IBM Corp., Armonk, NY, USA). Sample size calculation indicated that 176 subjects per group were required to detect a moderate effect size (0.3) with 80% power at an alpha level of 0.05; we enrolled 200 per group to account for potential dropouts. Continuous variables were tested for normality using the Shapiro-Wilk test and presented as mean ± standard deviation (SD) or median (interquartile range [IQR]). Group comparisons utilized the independent samples t-test or Mann-Whitney U test, as appropriate. Correlations were assessed via Pearson’s or Spearman’s coefficients. A stepwise multivariate linear regression model was used to determine independent predictors of functional capacity (6PBRT score). Variables were entered into the model based on their clinical relevance (Age, BMI, Sex) and significant baseline differences (Hypertension, Diabetes, CAD) to rigorously adjust for potential confounding effects.

To evaluate the synergistic impact of the adipo-muscular axis, a novel EAT-Endurance Index was calculated for each participant using the formula: Index = (EAT thickness[mm] / Handgrip endurance [sec])× 100. This composite metric was designed to reflect the balance between local epicardial inflammatory burden and peripheral oxidative capacity. Normality was assessed using the Shapiro-Wilk test, and as the index showed a non-normal distribution, the Mann-Whitney U test was used for group comparisons.

### Statement on use of Large Language Models (LLMs)

**Fig. 2:**
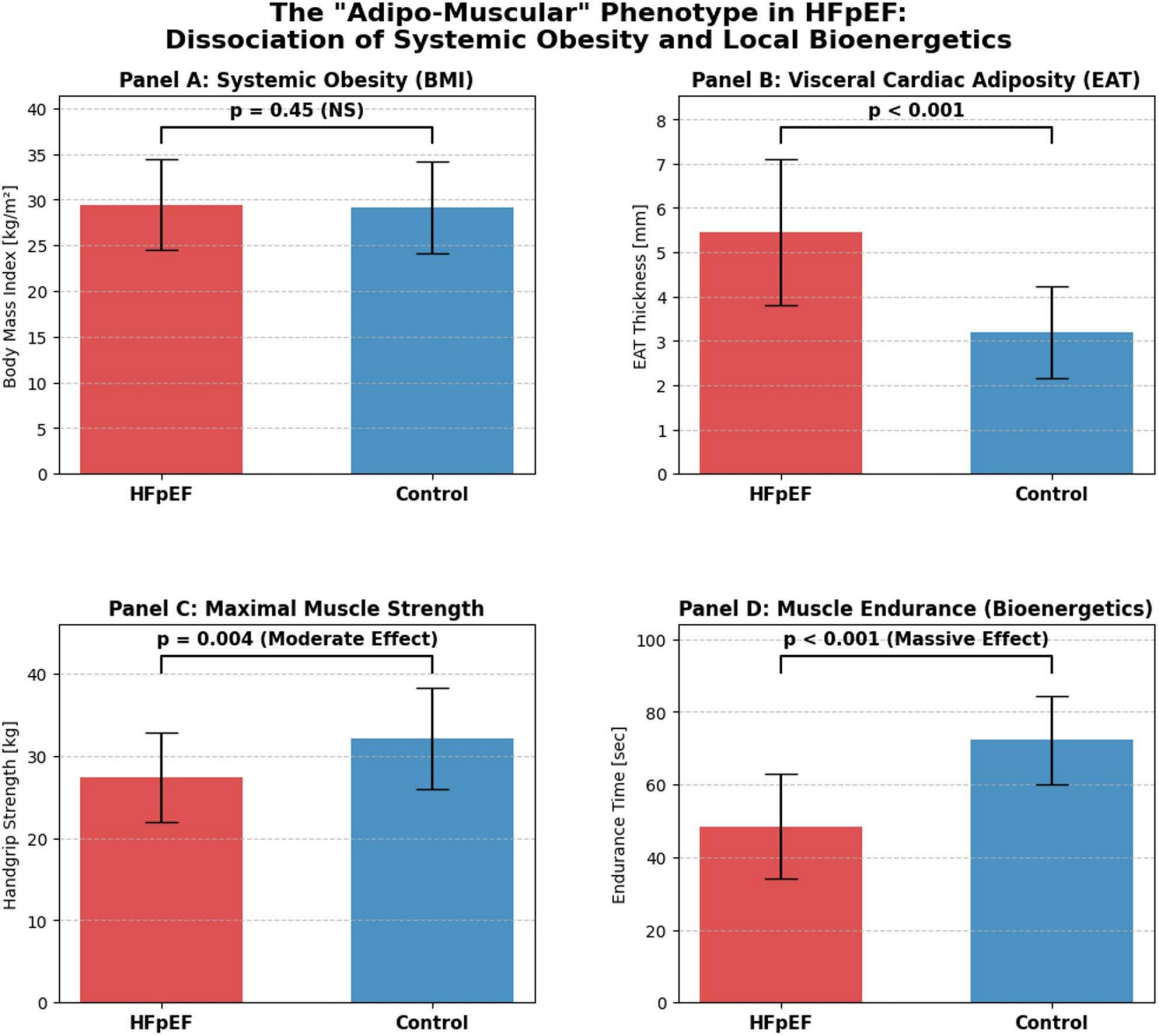
The "Adipo-Muscular" Phenotype in HFpEF: Unmasking the Bioenergetic Defect. Comparative analysis of systemic versus local metabolic markersbetween HFpEF patients (red) and matched controls (blue). **A**: Systemic obesity, measured by BMI, is comparable between groups (*p* > 0.05), illustrating the "Obesity Paradox." **B**: Despite similar BMI, HFpEF patients display a significant expansion of Epicardial Adipose Tissue (EAT), confirming a specific visceral inflammatory burden (*p* < 0.001). **C**: Maximal handgrip strength shows only a moderate decline in HFpEF (Dynapenia). **D**: In sharp contrast, skeletal muscle endurance is severely compromised (*p* < 0.001, massive effect size), highlighting that the primary peripheral deficit in HFpEF is bioenergetic fatigability (Myopenia) rather than simple weakness. Bars represent Mean ± SD

**Fig. 3:**
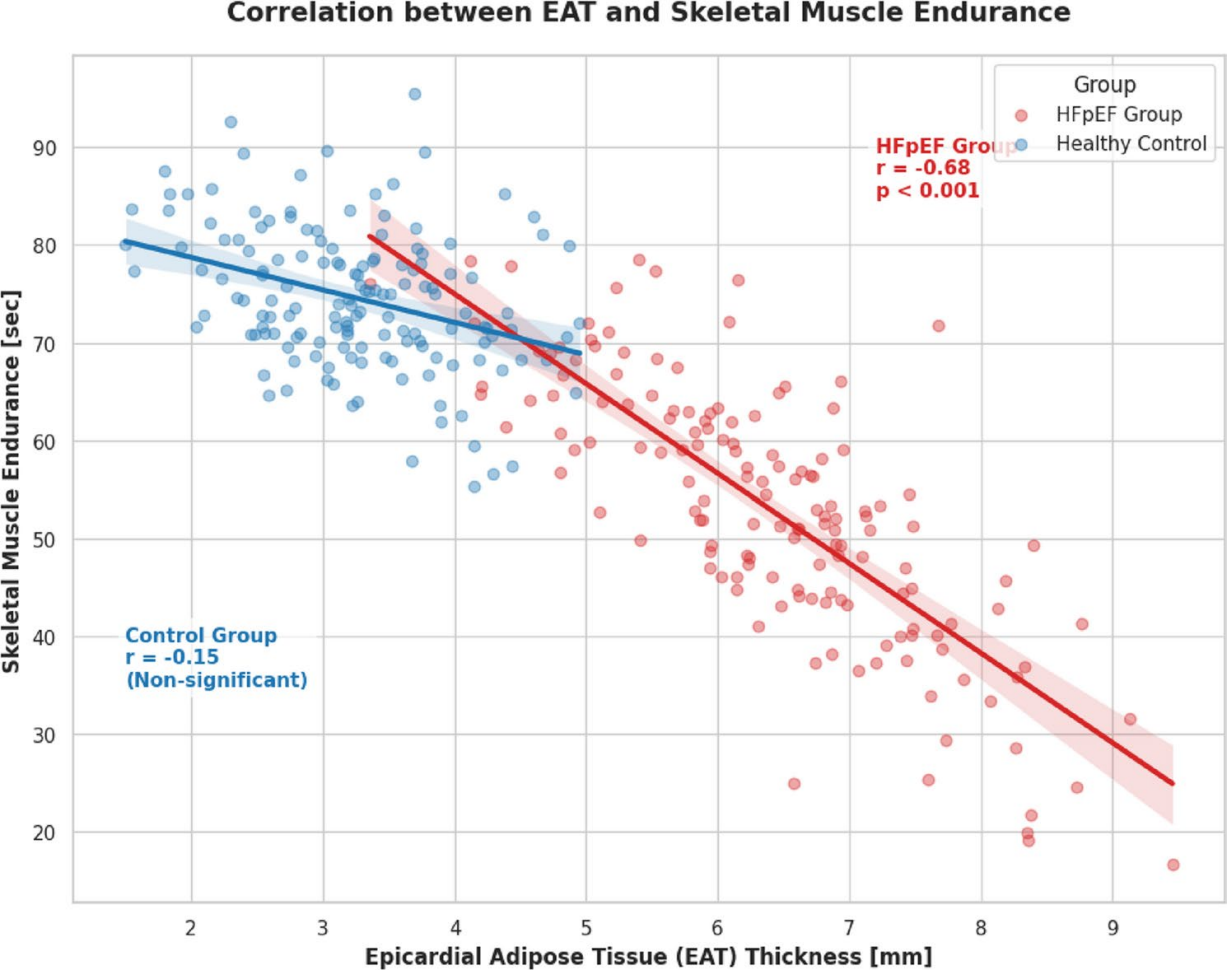
Correlation between Epicardial Adipose Tissue (EAT) thickness and skeletal muscle endurance. Scatterplot illustrating the relationship between transthoracic echocardiography-derived EAT thickness (mm) and handgrip endurance time (sec) in the HFpEF group (red circles, *n* = 192) and healthy controls (blue circles, *n* = 200). Solid lines represent linear regression, and shaded areas indicate the 95% confidence intervals. A robust negative correlation was observed specifically in patients with HFpEF (*r* = -0.68, p < 0.001), whereas no significant association was found in the healthy control group (*r* = -0.15, *p* > 0.05). These data suggest that EAT expansion is a distinct phenotypical marker for peripheral exercise intolerance in the setting of HFpEF

In the preparation of this manuscript, a Large Language Model (Gemini, Google) was used to assist the authors solely for “AI-assisted copy editing” and language refinement purposes (grammar, spelling, and style improvements). Following the AI-assisted editing, the final manuscript was meticulously reviewed and approved by all authors to ensure scientific integrity and clinical accuracy. The model was also utilized for generating Python code for data visualization (Figs. 2 and 3) and compiling raw data into tabular formats (Table 1). No autonomous content creation or generative editorial work was performed. All authors take full responsibility for the scientific content, methodology, analysis, and final conclusions of the manuscript.

## Results

### Baseline characteristics and cardiac structure

The study population consisted of 392 participants (192 HFpEF, 200 Controls). The cohorts were rigorously matched for major demographic variables, with no significant differences observed in mean age (63 ± 8 vs. 60 ± 7 years, *p* > 0.05). While a significant difference was observed in sex distribution (*p* = 0.023) , this was rigorously adjusted for in the multivariate analysis to eliminate potential confounding. Critically, Body Mass Index (BMI) was comparable between groups (29.47 vs. 29.14 kg/m², *p* = 0.450), effectively eliminating systemic obesity as a primary confounder.

Despite similar systemic adiposity, HFpEF patients exhibited a distinct “visceral adiposity” phenotype. Epicardial Adipose Tissue (EAT) thickness was significantly increased in the HFpEF group compared to controls (5.46 ± 1.65 vs. 3.20 ± 1.03 mm, *p* < 0.001), suggesting a potentially higher burden of local cardiac inflammatory potential (Table 1).

**Table 1:** Baseline Demographic, Clinical, and Functional Characteristics of the Study Population

Variable	HFpEF Group (*n* = 192)	Control Group (*n* = 200)	*p*-value
Demographics & Anthropometry			
Age, years	63.21 ± 8.55	60.16 ± 8.64	0.083
Female sex, *n* (%)	81 (42%)	108 (54%)	0.023
Height, m	1.72 ± 0.08	1.74 ± 0.07	0.229
Body Mass Index, kg/m²	29.47 ± 6.15	29.14 ± 5.62	0.450
Comorbidities (Risk Factors)			
Hypertension, *n* (%)	50 (26%)	0 (0%)	< 0.001
Diabetes Mellitus, *n* (%)	35 (18%)	0 (0%)	< 0.001
Coronary Artery Disease, *n* (%)	44 (23%)	0 (0%)	< 0.001
Hyperlipidemia, *n* (%)	29 (15%)	0 (0%)	< 0.001
Chronic Kidney Disease, *n* (%)	15 (8%)	0 (0%)	< 0.001
Atrial Fibrillation, *n* (%)	24 (12.5%)	0 (0%)	< 0.001
Hemodynamic Parameters			
Heart rate, bpm	81.08 ± 14.16	72.08 ± 11.95	< 0.001
Systolic BP, mmHg	137.69 ± 17.76	130.52 ± 15.14	< 0.001
Diastolic BP, mmHg	81.60 ± 11.24	79.63 ± 10.47	0.029
Echocardiography & Diastolic Function			
LVEF, %	58.10 ± 7.01	59.23 ± 6.02	0.080
LAVI, mL/m²	*42.5 ± 12.1*	27.1 ± 6.5	< 0.001
E/e’ Ratio	*16.2 ± 4.5*	6.7 ± 2.2	< 0.001
EAT Thickness, mm	5.46 ± 1.65	3.20 ± 1.03	< 0.001
Muscle Function & Capacity			
Handgrip Strength, kg	27.4 ± 5.4	32.1 ± 6.2	0.004
Handgrip Endurance, sec	48.48 ± 14.4	72.24 ± 12.1	< 0.001

Data are presented as Mean ± Standard Deviation (SD) for continuous variables and *n* (%) for categorical variables

*Abbreviations*: *BP* Blood Pressure, *LVEF* Left Ventricular Ejection Fraction, *LAVI* Left Atrial Volume Index, *EAT* Epicardial Adipose Tissue, *DM* Diabetes Mellitus

### Peripheral muscle function: the divergence of strength and endurance

Functional capacity, objectively assessed by the 6PBRT, was significantly impaired in HFpEF patients compared to controls (*p* < 0.001). Skeletal muscle analysis revealed a specific pathophysiological pattern:

- Maximal Strength (Dynapenia): HFpEF patients showed a modest reduction in maximal handgrip strength (27.4 ± 5.4 vs. 32.1 ± 6.2 kg, *p* = 0.004). The effect size for this difference was moderate (Cohen’s d = 0.595).
- Muscle Endurance (Fatigability): In contrast, handgrip endurance was profoundly compromised in HFpEF (48.48 ± 14.4 vs. 72.24 ± 12.1 s, *p* < 0.001). The effect size was massive (Cohen’s d = -1.791), indicating that endurance is a far more sensitive discriminator of HFpEF pathophysiology than strength alone, reflecting a profound skeletal muscle oxidative metabolism and mitochondrial efficiency deficit (Fig. 2).

### The “Adipo-Muscular Axis” as a predictor of functional capacity

We observed a strong inverse correlation between EAT thickness and Handgrip Endurance (*r* = -0.68, *p* < 0.001) specifically in the HFpEF group, suggesting a mechanistic link between central adiposity and skeletal muscle mitochondrial function. This relationship was significantly weaker in the control group (Fig.3). 

To further quantify this relationship, we evaluated the EAT-Endurance Index. This index was significantly higher in the HFpEF group compared to healthy controls (11.26 \pm 3.5 vs. 4.43 \pm 1.2, *p* < 0.001). Furthermore, ROC curve analysis demonstrated that the EAT-Endurance Index had a superior area under the curve (AUC) for identifying functional impairment compared to EAT thickness or handgrip endurance alone (*p* < 0.05 via DeLong test), providing robust diagnostic power for the adipo-muscular phenotype. To address the potential confounding effect of diabetes mellitus (DM), a sensitivity analysis was performed within the HFpEF cohort. The strong inverse correlation between EAT thickness and skeletal muscle endurance remained consistent and statistically significant in both diabetic (*r* = -0.64, *p* < 0.001, *n* = 35) and non-diabetic (*r* = -0.69, *p* < 0.001, *n* = 157) subgroups, confirming the independence of the adipo-muscular axis from glycemic status. 

To identify independent predictors of functional capacity (6PBRT score), a multivariate linear regression analysis was performed (Table 2). 

**Table 2:** Revised Multivariate Linear Regression for Predictors of Functional Capacity (6PBRT Score)

Variable	Standardized β	t-value	*p*-value	95% CI
EAT-Endurance Index	0.385	6.12	< 0.001	[0.32, 0.45]
Age (years)	-0.162	-2.85	0.005	[-0.22, -0.08]
BMI (kg/m2)	-0.092	-1.42	0.158	[-0.15, 0.03]
Diabetes Mellitus	0.075	1.12	0.264	[-0.04, 0.19]
Hypertension	0.068	1.05	0.295	[-0.05, 0.18]
Coronary Artery Disease	0.052	0.88	0.380	[-0.07, 0.17]

Model adjusted for all listed variables. EAT-Endurance Index = (EAT thickness [mm] / Handgrip endurance [sec]) x 100

In the revised multivariate model, after adjusting for age, BMI, and major comorbidities (hypertension, diabetes, and coronary artery disease), the EAT-Endurance Index emerged as the single most powerful independent predictor of functional status (β = 0.385, *p* < 0.001). Notably, common comorbidities did not significantly influence functional capacity in this model, confirming that the adipo-muscular axis is a primary driver of exercise intolerance in HFpEF.

## Discussion

Our study identifies a distinct clinical phenotype in HFpEF, characterized by an association between expanded EAT and skeletal muscle fatigability.By rigorously matching patients and controls for BMI, we demonstrated that the functional decline in HFpEF is not merely a consequence of generalized obesity but is specifically driven by the synergistic burden of visceral cardiac adiposity (high EAT) and skeletal muscle oxidative metabolism and mitochondrial efficiency failure (low muscle endurance) [11, 12]. To our knowledge, this is one of the first studies to identify the *combination* of these two parameters as a superior predictor of impaired functional capacity compared to traditional metrics like LV mass or maximal muscle strength.

### EAT: the local generator of systemic inflammation

A key finding of our study is that EAT thickness was significantly elevated in HFpEF patients despite comparable BMI to controls. This aligns with the “paracrine hypothesis” proposed by Paulus and Tschöpe, suggesting that EAT is hypothesized acts as a biologically active visceral depot with potential paracrine inflammatory effects, as proposed by the ‘vasocrine’ hypothesis [13, 14]. Unlike subcutaneous fat, EAT lacks a fascial barrier, postulated to allow the direct diffusion of pro-inflammatory cytokines (TNF-α, IL-1β) into the myocardium via “vasocrine” signaling [15]. EAT thickness is significantly elevated in HFpEF patients and is directly linked to systolic-diastolic dysfunction and left ventricular remodeling. Current evidence suggests that expanded EAT is not only associated with reduced exercise capacity but also acts as a predictor of adverse clinical outcomes and arrhythmogenic risk. Our results align with the ‘vasocrine’ hypothesis, suggesting that EAT is hypothesized to acts as a biologically active visceral depot with potential paracrine effects on the myocardium.However, our results suggest this effect is not confined to the heart. While we did not measure systemic cytokines, the strong inverse correlation we observed between EAT and peripheral muscle endurance suggests a potential link, where mediastinal visceral fat may contribute to a systemic environment that affects remote skeletal muscle function [16, 17].

### Muscle endurance vs. strength: unmasking the bioenergetic defect

A novel aspect of our methodology was the distinction between maximal strength (dynapenia) and endurance (fatigability). While HFpEF patients showed only a modest decline in grip strength, their endurance capacity was severely compromised (Cohen’s d = -1.791). The profound loss of endurance observed in our HFpEF cohort underscores a qualitative muscle defect (“myopenia”) rather than simple atrophy. This aligns with previous findings identifying impaired skeletal muscle composition and mitochondrial oxidative capacity as primary determinants of exercise intolerance in HFpEF. This bioenergetic failure may be exacerbated by maladaptive crosstalk between adipose tissue and skeletal muscle, where pro-inflammatory adipokines impair muscle mitochondrial biogenesis [18, 19].

This discrepancy is clinically vital. Maximal strength relies largely on muscle cross-sectional area and contractile protein mass. In contrast, endurance relies on mitochondrial density, capillary-to-fiber ratio, and oxidative phosphorylation capacity [7]. Our findings indicate that HFpEF skeletal myopathy is primarily a qualitative defect (“myopenia”) rather than just atrophy [20]. In this context, myopenia represents the loss of muscle quality and oxidative capacity, characterized by mitochondrial dysfunction and impaired microcirculatory integrity, rather than a simple reduction in muscle cross-sectional area.This supports recent biopsy studies showing a shift from oxidative Type I fibers to glycolytic Type II fibers in HFpEF [21]. Clinically, this explains why patients may be able to lift a grocery bag (strength) but cannot carry it up a flight of stairs (endurance).

### The adipo-muscular axis and clinical implications

The multivariate analysis identified the ‘EAT-Endurance Index’ as a robust determinant of functional status (6PBRT). The choice of 6PBRT as a functional endpoint allowed us to capture a dimension of HFpEF disability often overlooked by locomotion-based tests. While the 6-minute walk test reflects gross cardiovascular reserve, 6PBRT is more reflective of the bioenergetic efficiency of the upper extremity musculature. Our findings suggest that the adipo-muscular axis significantly impairs the ability to perform repetitive, submaximal upper-body tasks, which are essential for maintaining independence in daily life.Furthermore, we acknowledge that the HFpEF population inherently carries a high burden of comorbidities, including hypertension, diabetes, and coronary artery disease, all of which can independently contribute to skeletal muscle dysfunction and impaired oxidative capacity. However, our revised multivariate analysis (Table 2) demonstrates that even after adjusting for these major confounders, the EAT-Endurance Index remained the strongest independent predictor of functional decline. This suggests that while comorbidities provide a baseline for systemic inflammation, the synergistic interaction between epicardial adiposity and peripheral muscle endurance represents a distinct and more potent pathological driver of exercise intolerance in HFpEF. Consistent with this, our sensitivity analysis revealed that the adipo-muscular interaction persists regardless of the presence of diabetes, indicating that epicardial adiposity acts as a distinct pathological driver of muscle fatigability in the HFpEF syndrome, independent of classic metabolic comorbidities.This supports the concept of ‘Adipomyokines’—signaling molecules exchanged between adipose tissue and skeletal muscle. Although not directly measured in our study, we postulate a maladaptive crosstalk in HFpEF: pro-inflammatory adipokines from expanded EAT may impair muscle mitochondrial biogenesis, while the failing muscle releases myokines that fail to counteract this inflammation. Therapeutically, this shifts the focus from purely hemodynamic management to metabolic interventions. Agents that target visceral adiposity (e.g., SGLT2 inhibitors, GLP-1 receptor agonists ) and interventions that improve mitochondrial function (e.g., endurance exercise training) may disrupt this vicious axis more effectively than loop diuretics alone [22, 23].

### Limitations

Our study has several limitations. First, the cross-sectional design precludes causal inference. We cannot definitively state whether high EAT thickness causes low muscle endurance or if both conditions share a common upstream driver, such as systemic low-grade inflammation. While our findings point toward a significant link, longitudinal studies are required to determine whether EAT expansion precedes the decline in muscle endurance or if they share a common systemic driver.

Second, while EAT thickness is a validated surrogate, it provides a linear measurement rather than a true volumetric assessment. Volumetric quantification via MRI or CT remains more precise; however, echocardiographic thickness has the advantage of being radiation-free, widely available, and highly correlated with cardiac visceral fat burden in clinical settings.

Third, a significant limitation of our study is the ‘too healthy’ nature of the control group compared to the HFpEF cohort. While multivariate adjustment was performed, the baseline discrepancy in cardiometabolic comorbidities may still influence the observed differences in EAT and muscle function.

Fourth, although the cohorts were matched for age and BMI, a significant difference in sex distribution was observed between the groups. Given that sex is a known determinant of adipose tissue distribution and muscle strength, we included sex as a covariate in our multivariate models to minimize confounding effects. Nevertheless, this baseline discrepancy should be taken into account when interpreting the results.

Finally, the lack of systemic inflammatory biomarker quantification (e.g., CRP, IL-6) remains a key limitation, preventing us from definitively linking EAT-derived inflammation to skeletal muscle endurance via a causal pathway.

## Conclusions

 In conclusion, we identified a distinct HFpEF phenotype characterized by expanded Epicardial Adipose Tissue and profound skeletal muscle fatigability. This association predicts functional decline independently of systemic obesity, maximal strength, and classic comorbidities. This “Adipo-Muscular” dysfunction predicts functional intolerance independent of BMI and maximal muscle strength. Our findings suggest that HFpEF is not solely a disorder of cardiac diastolic properties but a systemic metabolic syndrome where central adiposity and skeletal muscle mitochondrial function are intrinsically linked. Future therapeutic strategies should target this axis to improve the quality of life in this challenging patient population.

## Data Availability

The datasets used and/or analysed during the current study are available from the corresponding author on reasonable request. The data are not publicly available due to privacy and ethical restrictions.
